# Hypoxia and IF_1_ Expression Promote ROS Decrease in Cancer Cells

**DOI:** 10.3390/cells7070064

**Published:** 2018-06-21

**Authors:** Gianluca Sgarbi, Giulia Gorini, Francesca Liuzzi, Giancarlo Solaini, Alessandra Baracca

**Affiliations:** 1 Department of Biomedical and Neuromotor Sciences, Laboratory of Biochemistry and Mitochondrial Pathophysiology, University of Bologna, Bologna 40126, Italy; giulia.gorini@unifi.it (G.G.); francesca.liuzzi@studio.unibo.it (F.L.); giancarlo.solaini@unibo.it (G.S.); alessandra.baracca@unibo.it (A.B.); 2 Department of Biomedical, Experimental, and Clinical Sciences “Mario Serio”, University of Florence, Florence 50121, Italy

**Keywords:** cancer cells, osteosarcoma, IF_1_, F_1_F_0_-ATPase, hypoxia, ROS, superoxide radical

## Abstract

The role of reactive oxygen species (ROS) in the metabolic reprogramming of cells adapted to hypoxia and the interplay between ROS and hypoxia in malignancy is under debate. Here, we examined how ROS levels are modulated by hypoxia in human cancer compared to untransformed cells. Short time exposure (20 min) of either fibroblasts or 143B osteosarcoma cells to low oxygen tension down to 0.5% induced a significant decrease of the cellular ROS level, as detected by the CellROX fluorescent probe (−70%). Prolonging the cells’ exposure to hypoxia for 24 h, ROS decreased further, reaching nearly 20% of the normoxic value. In this regard, due to the debated role of the endogenous inhibitor protein (IF_1_) of the ATP synthase complex in cancer cell bioenergetics, we investigated whether IF_1_ is involved in the control of ROS generation under severe hypoxic conditions. A significant ROS content decrease was observed in hypoxia in both IF_1_-expressing and IF_1_- silenced cells compared to normoxia. However, IF_1_-silenced cells showed higher ROS levels compared to IF1-containing cells. In addition, the MitoSOX Red-measured superoxide level of all the hypoxic cells was significantly lower compared to normoxia; however, the decrease was milder than the marked drop of ROS content. Accordingly, the difference between IF_1_-expressing and IF_1_-silenced cells was smaller but significant in both normoxia and hypoxia. In conclusion, the interplay between ROS and hypoxia and its modulation by IF_1_ have to be taken into account to develop therapeutic strategies against cancer.

## 1. Introduction

Reactive oxygen species (ROS) are critical chemicals in cells: at controlled concentrations, they function as second messengers mediating the responses of cells to various endogenous and exogenous signals [1], although at high concentrations ROS cause a redox imbalance and subsequent oxidative stress [2]. This induces cytotoxicity due to oxidation of lipids, proteins, and nucleic acids, particularly within mitochondria, where ROS are mainly produced at the level of redox centers of the respiratory chain [3,4]. Besides the respiratory chain Complexes I-III, cytoplasmic oxidoreductases can generate ROS as a by-product, including cyclooxygenase, uncoupled nitric oxide synthase [5], and xanthine oxidase [1]. In addition, membrane proteins such as β-nicotinamide adenine dinucleotide 2′-phosphate (NADPH) oxidases deliberately produce hydrogen peroxide [6]. Low levels of oxygen in tissues (hypoxia) arise in both normal development and different pathophysiological conditions where limited oxygen supply is frequently caused by a defective vasculature. Such conditions include ischemic disorders, atherosclerosis, inflammatory diseases, chronic obstructive pulmonary disease and cancer [7]. Hypoxia induces HIF-1α stabilization in cells (the subunit α of the hypoxia-inducible factor 1) that in turn triggers the activation of a cellular adaptive response that is mediated by HIF-1, the master regulator of transcription in hypoxia. Mitochondria are one of the main targets of this process since HIF-1 can induce pyruvate dehydrogenase kinase-1 (PDK1) activation, that hinders pyruvate dehydrogenase (PDH) activity, thus limiting the substrates availability to the Tricarboxylic Acids Cycle, and consequently to oxidative phosphorylation (OXPHOS) [8]. Other factors have been shown to contribute to OXPHOS activity decrease in cells exposed to hypoxia, including mitophagy and Complex I deactivation [9,10]. Nevertheless, our recent observations indicated that the adaptive response of normal human cells to hypoxia is strongly dependent on glucose availability. Indeed, in hypoxic human fibroblasts forced to rely on OXPHOS as the major source of ATP, the mitochondrial mass is almost completely preserved and the levels of OXPHOS enzymes are significantly increased; the contrary occurs when glucose is the main energy substrate of cells [9].

An important feature that is still under discussion concerns the level and the potential role of Reactive Oxygen Species (ROS) in cell adaptation to hypoxia. Several studies demonstrated that under hypoxia ROS production, mostly derived from the mitochondrial electron transport chain (ETC), increased in both normal and transformed cells [11,12,13,14], whereas others reported the opposite [15,16,17,18]. Two more peculiar papers are worth being mentioned: the first reported that hypoxia causes a ROS decrease in the mitochondrial matrix compartment of vascular smooth muscle cells, whereas it increases ROS production in the mitochondrial intermembrane space, which diffuse to the cytosol [18]. The second, a recent paper, reported a burst in superoxide radicals within the first 40 min of acute hypoxia declining afterwards to a level similar to normoxia in some cell types [19]. Therefore, a controversy exists regarding the effect of hypoxia on cellular ROS generation.

To address the study of the relationships and mechanisms connecting hypoxia, ROS, and cellular redox status in cancer and untransformed cells, we first validated a method to measure the levels of oxidant species in human primary fibroblasts adapted to decreased O_2_ tension (0.5% O_2_). The cellular ROS level dependence on oxygen tension has been investigated. The cancer cell model chosen was 143B osteosarcoma cell line being osteosarcoma the most common primary malignant bone tumor, that as a solid tumor is characterized by the presence of hypoxic areas. Finally, due to the critical role ascribed to the endogenous inhibitor protein (IF_1_) of the mitochondrial F_1_F_0_-ATPase in the regulation of cancer cell energy metabolism [20,21,22,23,24], we assayed whether IF_1_ is involved in the control of ROS generation in osteosarcoma cells and whether ROS handling could distinguish normal from transformed cells.

## 2. Materials and Methods

### 2.1. Reagents

Bovine serum albumin, Dulbecco’s Modified Eagle Medium, glucose, glutamine, NaCl, N-Acetyl-L-Cysteine (NAC), phenylmethylsulfonyl fluoride (PMSF), protease inhibitors, pyruvate, SDS, sodium deoxycholate, *tert*-butylhydroperoxide (Luperox), tris/Cl, triton X-100, were all purchased from Sigma-Aldrich (St. Luis, MO, USA). CellROX Orange and MitoSOX Red were from (Thermo Fisher Scientific, Waltham, MA, USA).

### 2.2. Cell Culture

Fibroblasts were obtained from skin biopsies of four healthy individuals (9–35 years) after informed consent had been obtained. Cell lines were established and expanded in Dulbecco’s modified Eagle’s medium (DMEM) containing 25 mM glucose, 4 mM glutamine, 1 mM pyruvate, 100 U/ml penicillin, 100 mg/ml streptomycin, 0.25 mg/ml amphotericin B, and supplemented with 15% fetal bovine serum (FBS) (Thermo Fisher Scientific, Waltham, MA, USA). Fibroblasts were seeded at 8 × 10^3^ cells/cm^2^ in high glucose to favor adhesion, and, after 16 h, the medium was replaced with the experimental one containing 5 mM glucose. Controls and IF_1_-silenced clones derived from the 143B osteosarcoma cell line [24] were seeded at 3.5 × 10^4^ cells/cm^2^ in high glucose medium containing 10% FBS to favor adhesion, and, after 16 h, the medium was replaced with fresh medium. Routine mycoplasma tests were performed to ensure the absence of contamination. All the cell types were cultured simultaneously for up to 24 h in two different incubators; in a humidified atmosphere at 37 °C containing 5% CO_2_ and either atmospheric (21% O_2_; pO_2_ = 21 kPa) or low (0.5% O_2_; pO_2_ = 0.5 kPa) oxygen tension as previously reported [15].

### 2.3. Flow Cytometry Analysis

Flow cytometry determination of ROS, the most contributors to total cellular reactive oxidant species, and superoxide anion was performed using a MUSE cytometer (Merk Millipore, Darmstadt, Germany) after loading the cells with either 5 μM CellROX Orange or 5 μM MitoSOX Red [25,26,27], respectively. The cells were incubated with each dye for 30 min at 37 °C under both normoxia and hypoxia (0.5% O_2_) and wells were then washed with HBSS to remove any remaining unincorporated dye. The cells were rapidly trypsinized, diluted to the optimal density with HBSS supplemented with 10% FBS and immediately analyzed. The cell fluorescence intensity was measured using a 532 nm excitation and a 576/28 nm emission filter; a total of 10,000 events were acquired for each analysis. Top right quadrant post-analysis of the cellular fluorescence distribution (expressed as percent of total events) were performed by the Flowing software (Cell Imaging Core, Turku Centre for Biotechnology, University of Turku).

To validate the CellROX Orange as a proper probe for cellular ROS detection, 1 mM N-Acetyl-Cysteine (NAC) and 200 μM *tert*-butylhydroperoxide (Luperox) were added to cells as negative and positive controls, respectively.

### 2.4. Immunoblot Analysis

Cells maintained under either normoxia or hypoxia (0.5% O_2_) were lysed, and proteins separated by sodium dodecyl sulfate-polyacrylamide gel electrophoresis (SDS-PAGE) were blotted onto nitrocellulose membranes to perform a semiquantitative analysis according to Sgarbi et al. [28]. Blots of resolved proteins were incubated with both mouse monoclonal anti-HIF-1α, anti IF_1_ (12 kDa), anti F_1_F_0_ ATPase a-subunit (54 kDa) (Abcam, Cambridge, UK) and mouse monoclonal anti-β actin (42 kDa) (Sigma-Aldrich, St. Louis, MO, USA) primary antibodies. Beta-Actin was used as an internal standard. Immunodetection of primary antibody was carried out with secondary goat anti-mouse IgGH + L antibody (Life Technologies, Carlsbad, CA, USA) labelled with horseradish peroxidase. Chemiluminescent detection of the specific proteins was performed with the ECL Western Blotting Detection Reagent Kit (GE Healthcare, Waukesha, WI, USA) using the ChemiDoc MP system equipped with ImageLab software (BioRad, Hercules, CA, USA) to perform the densitometric scanning of the relative protein intensity.

### 2.5. Protein Determination

Protein concentration of samples was assessed by a method previously reported [29]. Essentially, cellular protein content was determined in presence of 0.3% (*v*/*v*) sodium deoxycholate, using bovine serum albumin as standard.

### 2.6. Data analysis

Results were analyzed by means of the one-way analysis of variance (ANOVA) with Bonferroni’s post-hoc test. Statistical analysis was performed by running the OriginPro 7.5 software (Origin-Lab Corporation, Northampton, MA, USA). Data are reported as mean ± SD of at least three independent experiments. A level of *p* ≤ 0.05 was selected to indicate statistical significance.

## 3. Results

### 3.1. Validation of CellROX Responsiveness in Detecting ROS Level Changes

**Figure 1:**
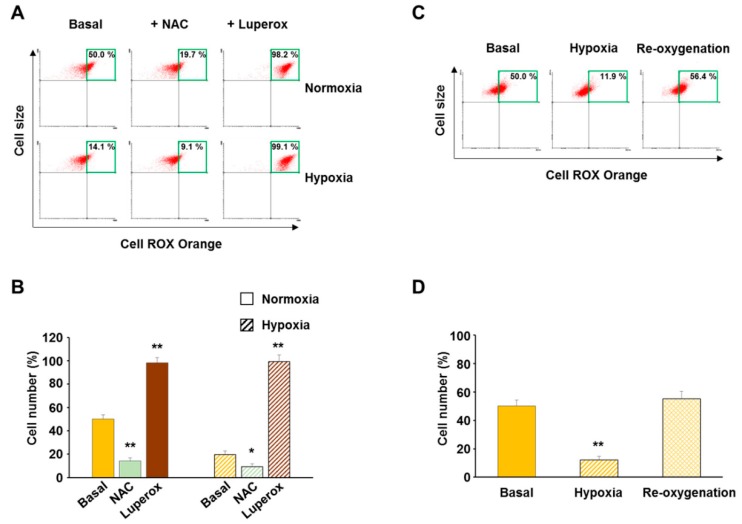
Validation of ROS detection by CellROX in human fibroblasts. (**A**) Typical top right quadrant (green-framed) analysis of cell fluorescence distribution as an index of ROS level. CellROX-loaded fibroblasts were analyzed following the exposure to 1 mM NAC or 200 μM Luperox, under both normoxia and hypoxia (6 h). (**B**) Quantitation of high fluorescent cells as an index of ROS content. (**C**,**D**) Fluorescence of CellROX-loaded control cells exposed to 4 h hypoxia followed by 4 h re-oxygenation. Data are means ± SD of three independent experiments, each carried out on four different cell lines. * *p* ≤ 0.05 and ** *p* ≤ 0.01 indicate the statistical significance of data compared to basal conditions.

Reactive oxygen species are important chemical intermediates in biological systems, playing a dual role as either intracellular messengers in physiological functions or detrimental molecules when their generation exceeds the cell capability to control it. Due to the high reactivity, the very short life span and the extremely low concentration of cellular ROS make their assessment critical. Several recent reviews addressed the issue and compared novel approaches with commonly used methods to assay ROS in cells [30,31,32]. We identified the new oxidative stress-sensitive dye CellROX Orange as a suitable and sensitive probe to investigate ROS level changes in human fibroblasts. Indeed, with the aim to assess the oxidative status of both normal and cancer cells in response to either acute or chronic hypoxia, we tested the fluorescence responsiveness of the probe to either tert-butylhydroperoxide (Luperox), as a positive control, or N-acetyl-L-cysteine, as a negative control, in primary human fibroblasts. Flow cytometry top right quadrant analysis of cell fluorescence distribution (expressed as percent of total events) allows to evaluate changes in cellular ROS levels. Under normoxia (6 h), the cells exposure to either 1 mM NAC or 0.2 mM Luperox before loading the probe, resulted in a change of the high fluorescence cells (top right quadrant cells), with a mean of nearly 20% and 100%, respectively, compared to basal conditions (Figure 1A,B). Under hypoxia (0.5% O_2_), the high fluorescence cells dropped to a mean residual 20% under basal condition and the exposure to NAC further decreased ROS levels to nearly 10%. Consistently, the presence of Luperox determined a strong increase of high fluorescence cells showing values similar to those observed in normoxia (Figure 1A,B). To further support the use of the CellROX fluorescent dye, we exposed fibroblasts to 4 h hypoxia followed by 4 h re-oxygenation. As expected, hypoxia-adapted fibroblasts exposed to 21% O_2_ reversed the high fluorescence cell percentage to the higher basal level (Figure 1C,D) showing that cellular ROS level changes were strictly related to oxygen tension.

### 3.2. Hypoxia Decreased ROS Level in Both Normal and Cancer Cells

**Figure 2:**
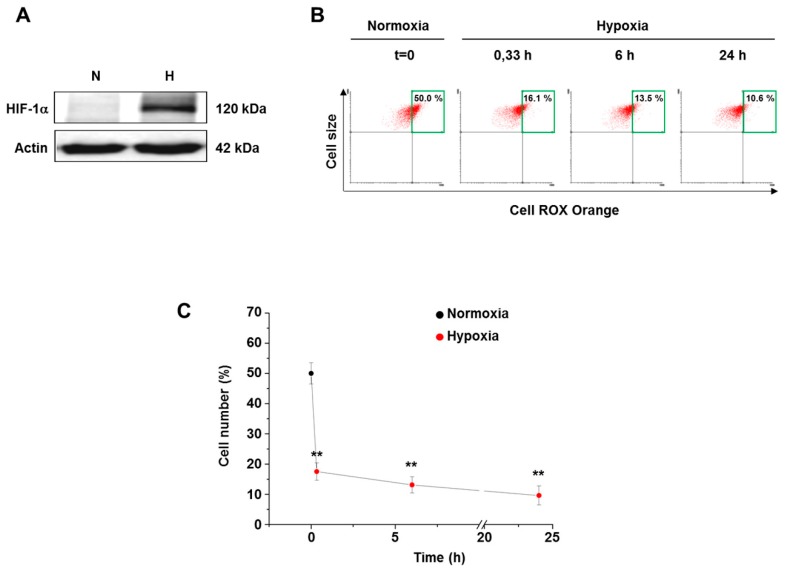
ROS level in human fibroblasts grown under hypoxia. (**A**) HIF-1α level determined upon 6 h exposure of fibroblasts to either normoxia or hypoxia. (**B**) Representative top right quadrant analysis of CellROX-loaded cells maintained in either normoxia or hypoxia (0.5% O_2_) up to 24 h. (**C**) Scatter graph showing the time-dependence of cellular ROS level decrease during 24 h hypoxic exposure. Data are means ± SD of three independent experiments, each carried out on four different cell lines. ** *p* ≤ 0.01 indicates the statistical significance of data compared to normoxia.

Following the CellROX Orange cell loading, we assayed the fluorescence distribution of either normal or transformed cells adapted to hypoxia at different time points up to 24 h. We first confirmed that 0.5% oxygen tension stabilizes HIF-1α in normal human fibroblasts and hence activates the HIF-1-dependent hypoxia signaling pathways (Figure 2A). Under this condition, a sharp ROS level decrease was detected following 20 min hypoxic exposure of fibroblasts, being the mean high fluorescence cells percentage nearly 20% compared to the 50% normoxic basal value (Figure 2B,C). Maintaining cells up to 24 h under hypoxia resulted in a further consistent and progressive decline of cellular ROS levels (nearly 10% top right quadrant cells).

**Figure 3:**
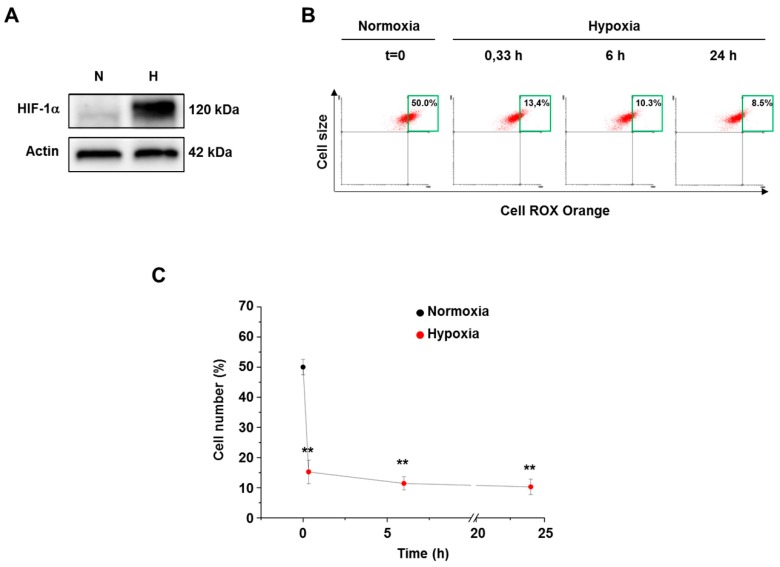
ROS level in osteosarcoma cells grown under hypoxia. (**A**) HIF-1α level determined upon 6 h exposure of osteosarcoma cells to either normoxia or hypoxia. (**B**) Representative top right quadrant analysis of CellROX-loaded cells maintained in either normoxia or hypoxia (0.5% O_2_ ) up to 24 h. (**C**) Scatter graph showing the time-dependence of cellular ROS level decrease during 24 h hypoxic exposure. Data are means ± SD of three independent experiments. ** *p* ≤ 0.01 indicates the statistical significance of data compared to normoxia.

Exposure of osteosarcoma 143B cells to severe hypoxia (0.5% O_2_) revealed a strong HIF-1α stabilization (Figure 3A). However, hypoxia similarly affected ROS levels of normal and transformed cells as shown by the dependence of the high fluorescence cell percentages on the hypoxic exposure time (Figure 3B,C). Again, transformed cells showed a steep fall of ROS content after 20 min hypoxic exposure, as shown by cell fluorescence distribution. A further mild decline of the cellular ROS levels up to 24 h was detected. Incidentally, ROS levels of 24 h hypoxia-exposed transformed cells were not affected by growing cells in 25 mM glucose concentration. Since several authors reported that a reduction of ROS levels in hypoxia might follow an initial burst of ROS [19], we also assessed ROS levels by exposing CellROX-loaded cells to a hypoxic short time (10 min). This condition again induced a decline of ROS level compared to normoxia (nearly 12% top right quadrant cells).

### 3.3. The Inhibitor Protein IF_1_ Controlled ROS Cellular Level

**Figure 4:**
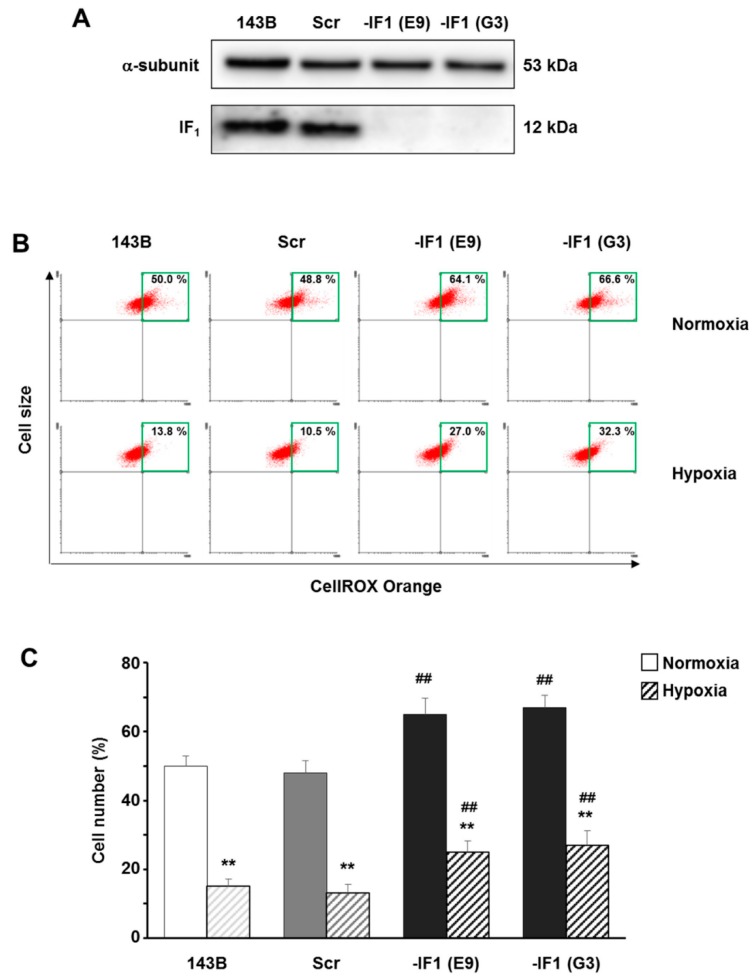
IF_1_ affects ROS level in osteosarcoma cells grown under either normoxia or hypoxia. (**A**) Immunoblot analysis of IF_1_ protein level in parental cells , scrambled and IF_1_-silenced clones (E9 and G3). (**B**) Representative top right quadrant analysis and (**C**) bars graph of ROS levels measured in all types of CellROX-loaded cells cultured in either normoxia or hypoxia (0.5% O_2_ ) for 24 h. Data are means ± SD of four independent experiments. ** *p* ≤ 0.01 and ## *p* ≤ 0.01 indicate the statistical significance of data compared to normoxia and to controls, respectively.

High ROS levels were detected in many types of cancers and were shown to be involved in both tumor development and progression. Moreover, according to recent studies, IF_1_ has been proposed to play a major role in the metabolic adaptation of cells during neoplastic transformation. Due to the emerging importance of ROS homeostasis and IF_1_ up-regulation in cancer cells, we hypothesized a putative role of IF_1_ in the modulation of cellular oxidative status. In order to address this issue, we explored the role of IF_1_ on the modulation of cellular ROS in both normoxia and hypoxia (0.5% O_2_) by silencing IF_1_ in 143 osteosarcoma cells. ROS level changes were assayed by using the CellROX Orange probe in both IF_1_-expressing cells (143B parental cell line and scrambled clones) and two stably IF_1_-silenced clones (Figure 4A), obtained as previously described [24]. A significant increase of ROS levels (about 65% top right quadrant cells) was detected in both IF_1_-silenced clones compared to controls, when cells were cultured at 21% oxygen tension for 24 h (Figure 4B,C). Although we observed a significant ROS content decrease in all types of cells under hypoxia, IF_1_-silenced clones still displayed higher ROS levels compared to controls.

### 3.4. IF_1_ Limited the Superoxide Anion Generation in Osteosarcoma Cells

**Figure 5:**
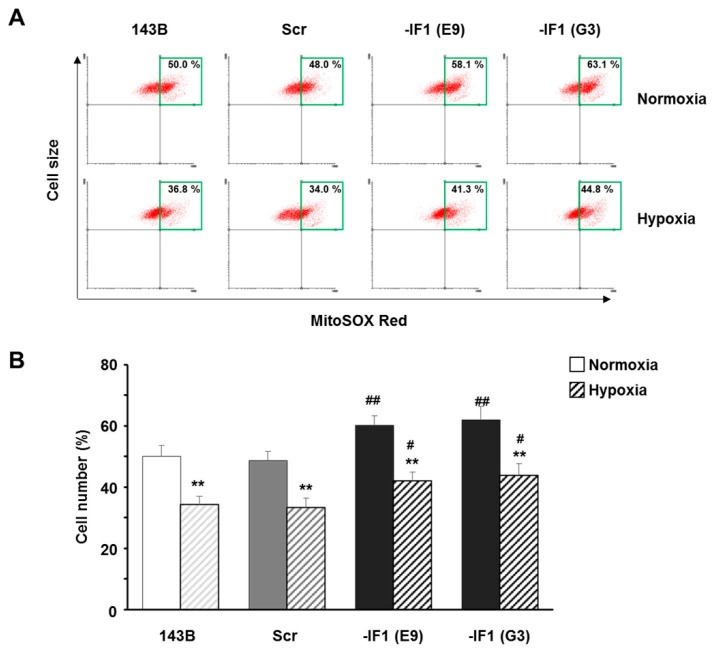
IF_1_ affects superoxide anion production in osteosarcoma cells grown under either normoxia or hypoxia. (**A**) Representative top right quadrant analysis and (**B**) bars graph of superoxide anion levels measured in MitoSOX Red-loaded parental (143B), scrambled (Scr) and IF_1_-silenced (E9 and G3) cells cultured in either normoxia or hypoxia (0.5% O_2_) for 24 h. Data are means ± SD of four independent experiments. ** *p* ≤ 0.01, indicates the statistical significance of data compared to normoxia; # *p* ≤ 0.05 and ## *p* ≤ 0.01, indicate the statistical significance of data compared to controls.

The higher ROS content, assessed in IF_1_-silenced cells compared to controls in both normoxia and hypoxia, prompted us to investigate whether these changes were associated with a different rate of mitochondrial superoxide anion production. To this aim, we performed experiments by using the specific and sensitive mitochondria-targeted superoxide probe MitoSOX Red [27]. Loading cells with the dye revealed a mild but significant increase of the superoxide anion production in IF_1_-silenced cells compared to controls, when cells were exposed to either normoxia (about 61 and 50% top right quadrant cells in IF_1_-silenced and control cells, respectively) or hypoxia (about 43 and 33% top right quadrant cells in IF_1_ silenced and control cells, respectively) for 24 h (Figure 5B). Furthermore, according to the cell fluorescence distribution parameter the superoxide production rate of all the hypoxic cells was significantly lower compared to normoxia (Figure 5A,B). Incidentally, the mitochondrial superoxide production rate of cells adapted to hypoxia showed a milder decrease compared to the marked drop of ROS content measured with the CellROX Orange probe, suggesting that in transformed cells, the superoxide anion is removed by the SODs quicker than hydrogen peroxide by peroxidases.

## 4. Discussion

In a hypoxic environment, often found in ageing human tissues and various diseases, mitochondrial metabolism of cells changes due to the alteration of membrane potential, OXPHOS complexes level, and OXPHOS rate, being all factors linked to energy substrates available to cells [9]. These changes certainly affect mitochondrial ROS homeostasis due to different rates of ROS production and/or removal; since ROS are the prevailing oxidants in cells, they are crucial players in determining the redox state of the cells. The latter has to be strictly controlled because it can affect cells [33]. Therefore, to properly design therapeutic interventions against cancer cells, it is of paramount importance to define both cellular level and parameters affecting ROS, considering in particular the oxygen concentrations experienced by that type of cell.

The main result of this study is that 143B osteosarcoma cells exposed to severe hypoxia present steady-state ROS levels lower than in normoxia, substantially behaving as non-transformed cells. We are aware that it is difficult to generalize the results due to the cell type individual response that depends on different levels and activity of endogenous antioxidants and detoxifying enzymes, and the different capabilities of cells to produce ROS through reactions in which oxygen is or is not a substrate. With regard to the osteosarcoma cells, it is well-established that solid tumors experience sharp decreases of O_2_ tension, due to the distance from the vascularization and the structural abnormalities characterizing the new-generated blood vessels [34]. According to the majority of the work published, ROS levels increase in cancer cells exposed to hypoxia [35], since the decreased O_2_ availability slows down the electron transport across the mitochondrial complexes, making the electrons able to leak out of the ETC and interact with O_2_, thus producing ROS [36]. In contrast, we found that when 143B osteosarcoma cells were exposed to 0.5% O_2_, a condition of severe hypoxia that mimics the very low O_2_ tension characterizing the most central areas of solid tumors, a substantial decrease of ROS occurred after 20 min. A decrease of the probe incubation time did not show any burst of ROS, in contrast with what was observed by others [19]. A further slight decline of ROS content was detected when cells were exposed to prolonged hypoxia (24 h). This behavior might be due to the 15% decrease of the mitochondrial mass recently observed in the osteosarcoma cells upon 24 h exposure [24]. As a whole, our results support the view that oxygen concentration is one of the main parameters determining ROS levels in cells, confirming our previous observations in normal cells exposed to a milder hypoxic condition [15].

It has been shown that mitochondria increase the production of the superoxide anion radical, a precursor of most other reactive oxygen species, when mitochondria hyperpolarization occurs. There is a general consensus on several reasons causing mitochondrial hyperpolarization, including an impaired activity of either the F_1_F_0_-ATPase and/or the Adenine Nucleotide Translocator and/or an impaired organization of the mitochondrial inner membrane, that can influence the redox centers and/or the redox reactions within the respiratory chain [37,38]. In a recent paper [20], we demonstrated that the silencing of the ATP synthase inhibitor factor, IF_1_, induces an increase of ΔΨ_m_ that pushed us to assay the production rate of the superoxide anion radical in mitochondria. We found that in normoxia, the superoxide level was slightly higher in IF_1_-silenced compared to IF_1_-expressing cells, as expected in accord with the higher ΔΨ_m_ measured in IF_1_-silenced cells. Surprisingly, in hypoxia, the superoxide level of IF_1_-silenced compared to IF_1_-expressing cells was still slightly higher, although ΔΨ_m_ of the two cell lines was quite similar. This implies that hypoxic conditions could stimulate mitochondrial superoxide radical production in IF_1_-silenced cells more than in IF_1_-expressing cells. The reason for this might be that IF_1_ deficiency causes an impairment of the mitochondrial cristae ultrastructure [21] to which a diminished OXPHOS super-complex assembly is associated [39], and this in turn can induce higher superoxide production [39,40]. However, the control exerted by IF_1_ on cellular oxidants is more marked when the whole ROS are assayed, as we did using the CellROX Orange.

## 5. Conclusions

The present study demonstrated that oxygen tension is one of the main factors affecting cellular ROS levels, and IF_1_ modulates the interplay between ROS and hypoxia in transformed cells. This might be relevant for the modulation of signaling pathways promoting cell survival, tumor progression, metastasis, and anticancer drug resistance [41,42], and has to be considered when designing innovative therapeutic approaches.
